# Delayed giant profunda femoris artery pseudoaneurysm caused by protruding screw after intertrochanteric fracture fixation: a case report

**DOI:** 10.1093/jscr/rjag265

**Published:** 2026-04-15

**Authors:** Andreja Dimic, David Matejevic

**Affiliations:** Faculty of Medicine, University of Belgrade, Belgrade, Serbia; Clinic for Vascular and Endovascular Surgery, University Clinical Center of Serbia, Belgrade, Serbia; Clinic for Vascular and Endovascular Surgery, University Clinical Center of Serbia, Belgrade, Serbia

**Keywords:** intertrochanteric fracture, pseudoaneurysm, profunda femoris artery, iatrogenic arterial injury

## Abstract

Iatrogenic femoral artery injury is a rare complication of hip fracture surgery but may cause serious morbidity if unrecognized. The profunda femoris artery is most commonly affected because of its close anatomical relationship to the proximal femur, and pseudoaneurysm formation is the typical delayed presentation. We report a 72-year-old woman with a progressively enlarging pulsatile mass in the right groin 6 months after fixation of an intertrochanteric fracture using a dynamic hip screw and plate. Imaging demonstrated a large profunda femoris artery pseudoaneurysm measuring 170 × 107 mm, likely caused by screw tips protruding beyond the medial femoral cortex. Open surgery was performed with thrombus evacuation, pseudoaneurysm sac resection, and oversewing of the injured profunda femoris artery. Recovery was uneventful. Although uncommon, profunda femoris artery injury should be suspected in patients with unexplained thigh swelling, pain, or anemia after hip fracture surgery. Early diagnosis and appropriate imaging are essential.

## Introduction

Iatrogenic vascular injury during surgery for proximal femoral fractures is rare but serious. The reported incidence is ~0.49%. Such injuries may lead to haemorrhage, pseudoaneurysm formation, distal ischemia, or other limb-threatening complications [1].

The profunda femoris artery is most frequently involved because of its proximity to the medial aspect of the proximal femur. Several mechanisms of injury have been described, including excessive drill penetration, protrusion of fixation screws beyond the medial cortex, and damage caused by retractors [2, 3]. Pseudoaneurysm formation is the most common manifestation and often presents with delayed, non-specific symptoms, which may lead to late diagnosis [4].

We present a case of a large delayed pseudoaneurysm of the profunda femoris artery that developed 6 months after fixation of an intertrochanteric fracture with a dynamic hip screw and plate, likely due to protruding screw tips.

## Case report

A 72-year-old woman was admitted with a progressively enlarging mass in the right groin region.

Six months earlier she had undergone surgical fixation of a right intertrochanteric femoral fracture. Shortly after surgery she noticed swelling in the right groin that gradually increased in size. During the month before admission the swelling enlarged rapidly and was accompanied by discomfort and pressure in the groin that made leg movement difficult.

**Figure 1:**
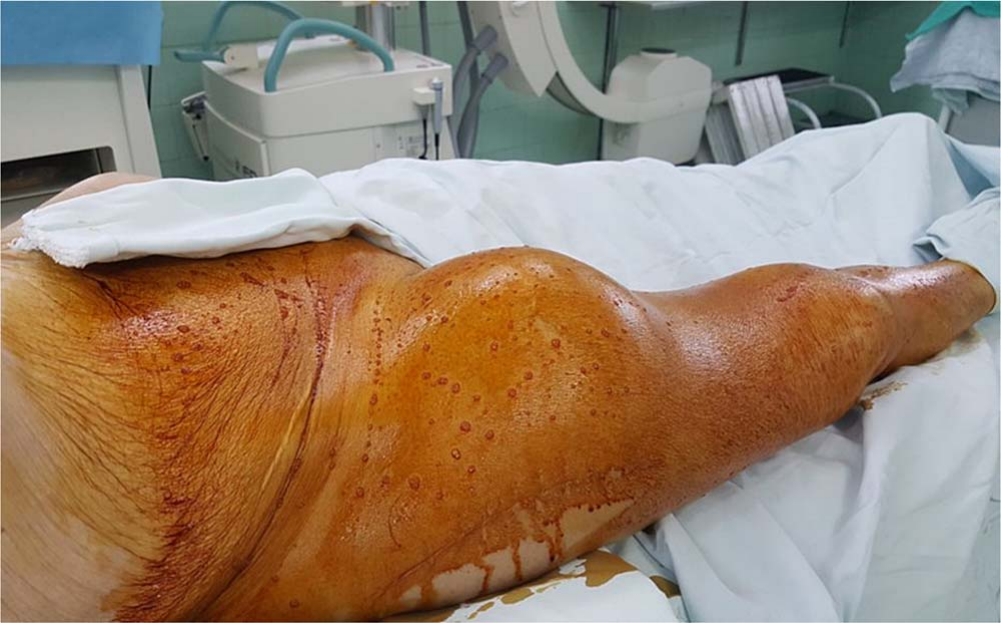
Clinical presentation of the patient. Large pulsatile mass visible in the right groin and proximal thigh region 6 months after fixation of an intertrochanteric femoral fracture.

On physical examination, a large tender pulsatile mass was palpable in the right groin (Fig. 1). Distal pedal pulses were present and symmetrical, and there were no signs of acute limb ischemia.

**Figure 2:**
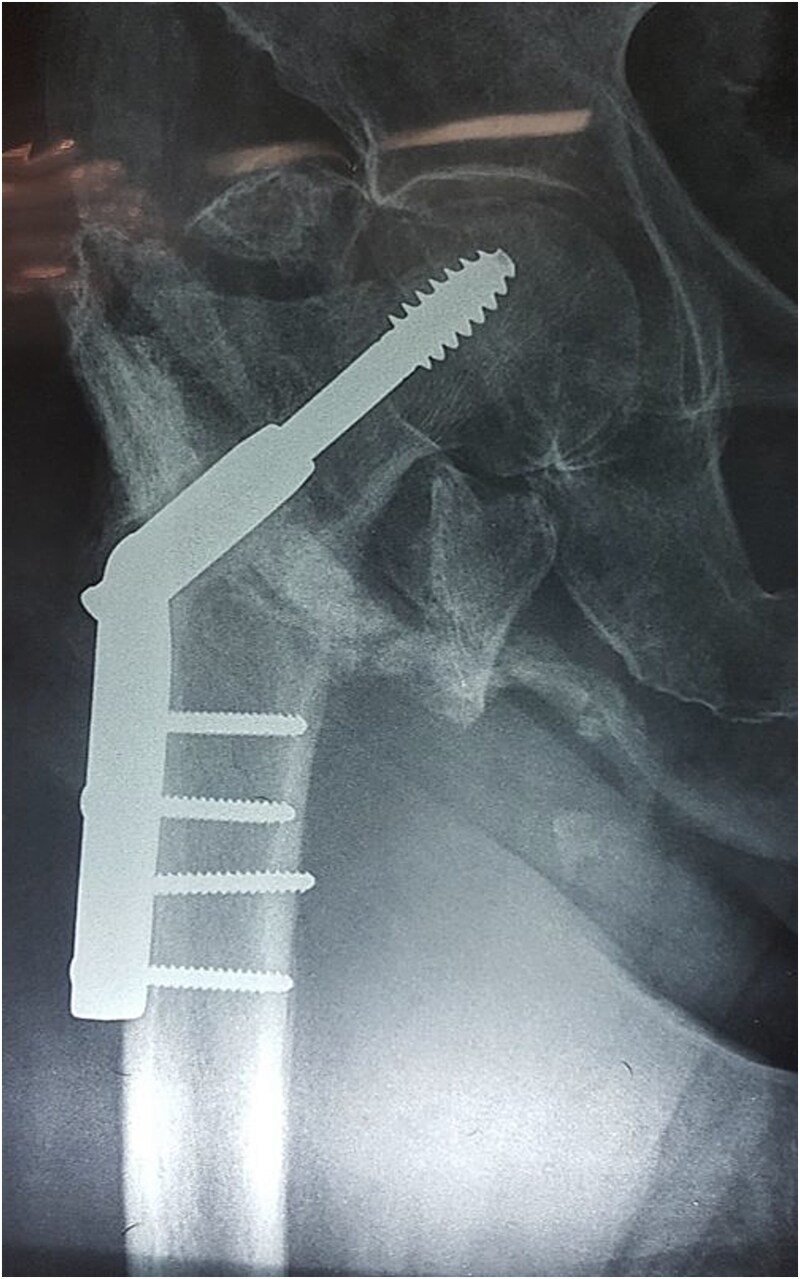
Plain radiograph of the right hip. Intertrochanteric femoral fracture previously treated with dynamic hip screw and plate fixation. Two cortical screws are visible protruding beyond the medial cortex of the femur, representing the probable mechanism of vascular injury.

Radiographs of the right hip demonstrated a previously treated intertrochanteric fracture with dynamic hip screw fixation. Two screws were observed protruding beyond the medial cortex of the femur (Fig. 2).

**Figure 3:**
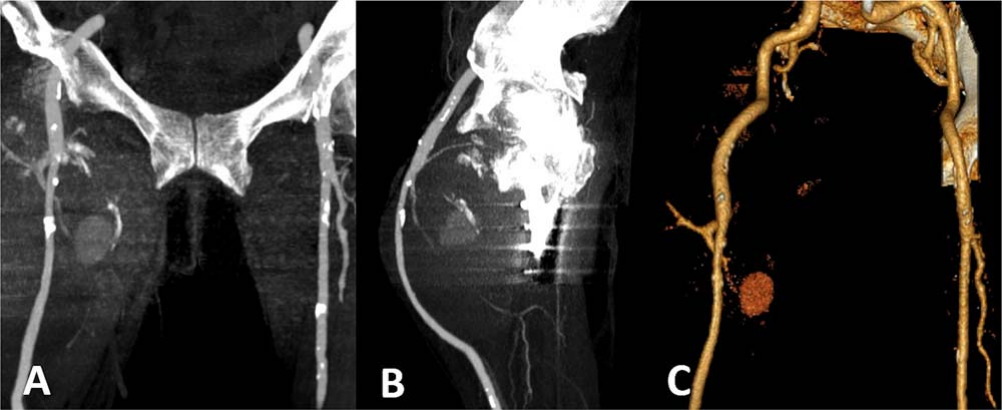
CT angiography. Large pseudoaneurysm (~170 × 107 mm) arising from the profunda femoris artery with partial thrombosis. The mass displaces the superficial femoral artery anteriorly. (A) Coronal CT image; (B) sagittal CT image; (C) three-dimensional reconstruction.

Colour duplex ultrasonography revealed a large heterogeneous mass measuring ~170 × 107 mm with partial thrombosis and residual arterial flow, consistent with pseudoaneurysm. Computed tomography (CT) angiography confirmed a large pseudoaneurysm arising from the profunda femoris artery and displacing the superficial femoral artery anteriorly (Fig. 3).

The patient was haemodynamically stable on admission. Laboratory tests demonstrated mild chronic anemia (haemoglobin 106 g/L).

**Figure 4:**
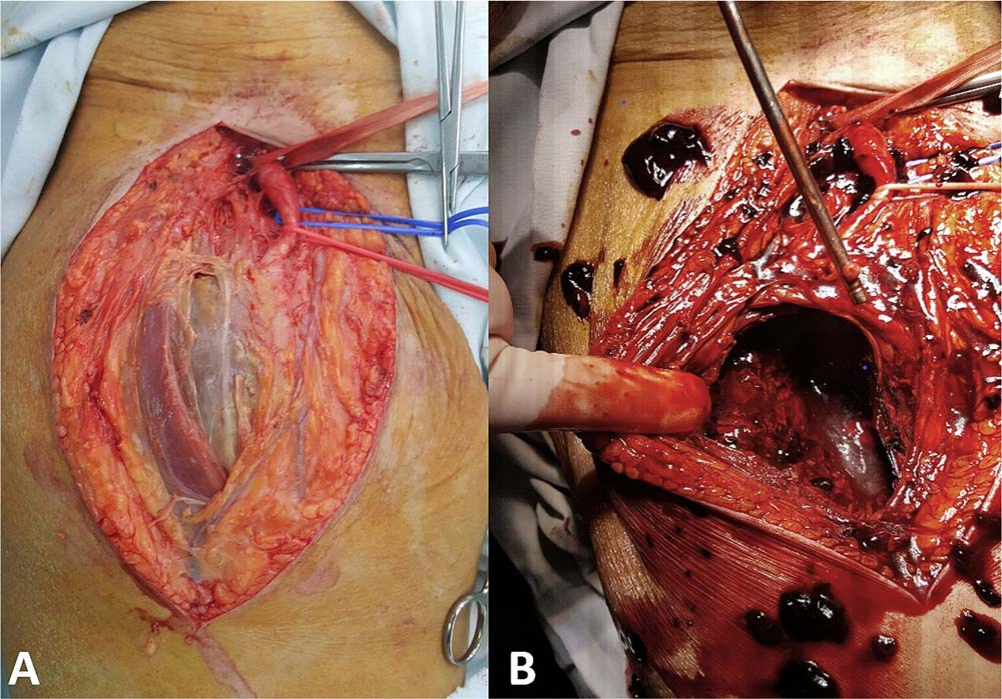
Intraoperative findings. (A) Exposure of the femoral vessels demonstrates the large pseudoaneurysm displacing the femoral bifurcation and superficial femoral artery and (B) the pseudoaneurysm sac was opened and the hematoma evacuated.

Because of the large size of the pseudoaneurysm and the associated mass effect, open surgical treatment was performed. Through a vertical groin incision the femoral vessels were exposed. Intraoperatively, the femoral bifurcation and superficial femoral artery were displaced anteriorly by the large pseudoaneurysm (Fig. 4).

**Figure 5:**
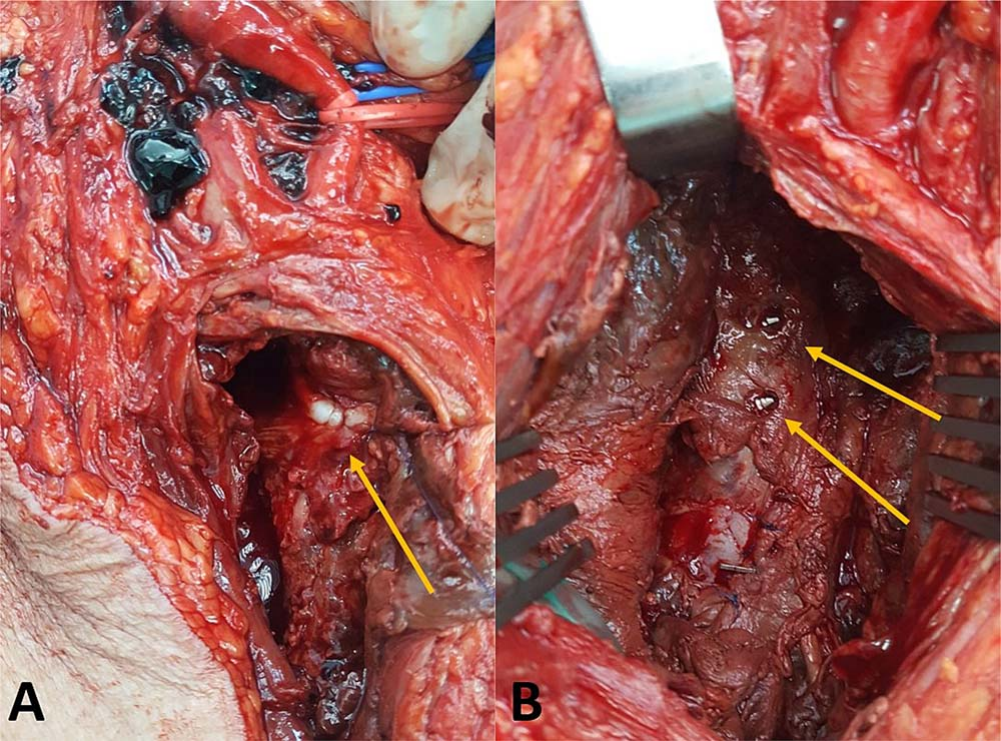
Intraoperative findings. (A) Oversewn laceration of the profunda femoris artery and (B) fixation screws protruding beyond the medial femoral cortex.

After systemic heparinization (100 IU/kg), the femoral arteries were clamped. The pseudoaneurysm sac was opened and a large amount of thrombus was evacuated. Active bleeding originating from a laceration of the profunda femoris artery was identified. As the superficial femoral artery was patent and provided adequate distal perfusion, the injured segment of the profunda femoris artery was oversewn. The presence of two screws protruding beyond the medial femoral cortex was also observed (Fig. 5). The pseudoaneurysm sac was resected and the wound closed in layers.

The postoperative course was uneventful and the patient recovered without complications.

## Discussion

Iatrogenic vascular injury associated with fixation of proximal femoral fractures is uncommon but may lead to serious complications [1]. Among the affected vessels, the profunda femoris artery is the most frequently involved, accounting for more than three-quarters of reported cases, followed by the superficial femoral artery [2]. This vulnerability is explained by the close anatomical relationship between the profunda femoris artery and the medial cortex of the proximal femur. During hip fracture surgery, positioning of the limb in adduction and internal rotation may further reduce the distance between the femur and adjacent vascular structures, increasing the risk of injury [2, 3].

Several mechanisms of iatrogenic arterial damage have been described, including excessive penetration by drill bits, injury caused by retractors, and protrusion of screws or fixation devices beyond the medial femoral cortex. Among these, screw protrusion is a well-recognized cause of delayed vascular injury because continuous mechanical irritation or gradual erosion of the arterial wall may eventually result in pseudoaneurysm formation [4–6].

Pseudoaneurysm is the most common clinical manifestation of vascular injury after hip fracture surgery. However, diagnosis is frequently delayed because the profunda femoris artery lies deep within the thigh musculature and early symptoms may be subtle. Patients may present with progressive thigh swelling, pain, anemia, or an enlarging pulsatile mass. These symptoms are often non-specific and may initially be attributed to postoperative hematoma or soft-tissue swelling related to the fracture [7]. In a series reported by Tiwary *et al.,* the mean time to presentation of pseudoaneurysm following orthopedic procedures was ~4 months, with patients typically presenting with progressive thigh swelling [4]. In our case, the pseudoaneurysm was diagnosed 6 months after the initial surgery and had reached a size of 170 × 107 mm.

If left untreated, pseudoaneurysms may lead to severe complications including rupture, distal ischemia, venous thrombosis, nerve compression, or compartment syndrome [8–10]. Early diagnosis is therefore essential. Colour duplex ultrasonography is usually the first diagnostic modality because it is noninvasive and readily available, while CT angiography provides detailed anatomical information and is useful for treatment planning [4, 6].

Management options include both open surgical repair and endovascular treatment. Endovascular techniques such as coil embolization, covered stent placement, or percutaneous thrombin injection are increasingly used, particularly in smaller pseudoaneurysms or in patients with high surgical risk [7, 11, 12]. However, large pseudoaneurysms associated with significant mass effect may be more appropriately treated with open surgery, which allows evacuation of thrombus and direct control of the bleeding vessel.

In the present case, the large size of the pseudoaneurysm and the presence of substantial thrombus favored open surgical management. Intraoperative findings together with radiographic evidence of screw protrusion beyond the medial femoral cortex strongly suggest that chronic arterial wall erosion caused by the screw tips was the mechanism of injury. The initial arterial damage likely resulted in a contained hematoma that gradually evolved into a pseudoaneurysm.

This case highlights the importance of careful surgical technique and avoidance of screw protrusion beyond the medial femoral cortex during fixation of proximal femoral fractures, as well as the need for a high index of suspicion when patients present with unusual postoperative swelling or anemia.
